# Jersey steer ruminal papillae histology and nutrigenomics with diet changes

**DOI:** 10.1111/jpn.13189

**Published:** 2019-09-04

**Authors:** Taylor E. Novak, Sandra L. Rodriguez‐Zas, Bruce R. Southey, Jessica D. Starkey, Ricardo M. Stockler, Gastón F. Alfaro, Sonia J. Moisá

**Affiliations:** ^1^ Department of Animal Sciences Auburn University Auburn AL USA; ^2^ Department of Animal Sciences University of Illinois Urbana IL USA; ^3^ Department of Poultry Science Auburn University Auburn AL USA; ^4^ College of Veterinary Medicine Auburn University Auburn AL USA

**Keywords:** bovine, diet change, gene expression, nutrigenomics, rumen epithelium

## Abstract

The transition from a high forage to a high concentrate diet is an important milestone for beef cattle moving from a stocker system to the feedlot. However, little is known about how this transition affects the rumen epithelial gene expression. This study assessed the effects of the transition from a high forage to a high concentrate diet as well as the transition from a high concentrate to a high forage diet on a variety of genes as well as ruminal papillae morphology in rumen fistulated Jersey steers. Jersey steers (*n* = 5) were fed either a high forage diet (80% forage and 20% grain) and transitioned to a high concentrate diet (20% forage and 80% grain) or a high concentrate diet (40% forage and 60% grain) and transitioned to a high forage diet (100% forage). Papillae from the rumen were collected for histology and RT‐qPCR analysis. Body weight had a tendency for significant difference (*p* = .08). Histological analysis did not show changes in papillae length or width in steers transitioning from a high forage to a high concentrate diet or vice versa (*p > *.05). Genes related to cell membrane structure (*CLDN1, CLDN4, DSG1*), fatty acid metabolism (*CPT1A, ACADSB*), glycolysis (*PFKL*), ketogenesis (*HMGCL, HMGCS2, ACAT1*), lactate/pyruvate (*LDHA*), oxidative stress (*NQO1*), tissue growth (*AKT3, EGFR, EREG, IGFBP5, IRS1*) and the urea cycle (*SLC14A1*) were considered in this study. Overall, genes related to fatty acid metabolism (*ACADSB)* and growth and development (*AKT3* and *IGFBP5*) had a tendency for a treatment × day on trial interaction effect. These profiles may be indicators of rumen epithelial adaptations in response to changes in diet. In conclusion, these results indicate that changes in the composition of the diet can alter the expression of genes with specific functions in rumen epithelial metabolism.

## 1 INTRODUCTION

With the rumen comprising approximately 70% of an adult ruminant's gut space (Church, 1988) and being responsible for up to 85% of the absorption of certain nutrients (Bannink et al., 2008), the rumen epithelial transcriptome is a good target for nutrigenomic research (Neeha & Kinth, 2013). There are several transcriptome studies about rumen epithelium adaptations to low‐grain diet, high‐grain diets or after induction of subacute ruminal acidosis (Kern et al., 2016; Li, Gelsinger, Edwards, Riehle, & Koch, 2019; Steele, Vandervoort, et al., 2011; Zhang, Zhu, & Mao, 2016) that we used as references to select the targeted genes for this study.

The rumen epithelium can alter its tissue mass in a variety of ways, by changing the degree of proliferation of the fraction of basal cells, changing cell division rates and adjusting cell transit times as a response to a dietary change from a roughage‐based to a concentrate‐based diet (Goodlad, 1981). In a previous study, after 4 weeks of continuous or transient high‐grain feeding, alterations in the gene expression profiles in the rumen epithelium compared with baseline could still be observed after a recovery period of 8 weeks (Petri, Wetzels, Qumar, Khiaosa‐Ard, & Zebeli, 2019). In order to enhance understanding of ruminal epithelial cell metabolism and proliferation during transition from different diets, several genes involved in different metabolic processes needs to be evaluated. We have used RT‐qPCR as a tool to study the effects of varying diets on targeted gene expression. Our hypothesis is that changing the diets by increasing or decreasing the amount of grain proportion will produce alterations on the expression level of genes related to cell membrane structure, tissue growth, fatty acid metabolism, glycolysis, ketogenesis, pyruvate/lactate metabolism, oxidative stress and urea cycle in the rumen epithelium. Our objective was trying to identify the biological processes more impacted in the rumen epithelium of Jersey steers, after periodic changes in the proportion of forage and grain in their diets throughout time. Better understanding of how expression of various genes in the rumen epithelium can be impacted by the alteration of nutrients provided may lead to improvements in productivity and feed efficiency of meat producing animals.

## 2 MATERIALS AND METHODS

### 2.1 Animals and sampling

All the procedures for this study were conducted in accordance with a protocol approved by the Institutional Animal Care and Use Committee of Auburn University (IACUC Protocol #2016‐2993). Five, approximately, two‐year‐old, rumen‐cannulated Jersey steers (between 370 and 420 kg of body weight) from Auburn University's College of Veterinary Medicine were used in this study. The assignment of steers to treatments was randomized across body weights such that the steers were ranked by weight, and the treatments were assigned in alternating fashion across the ranks. Animals were housed in a controlled environment room equipped with heating ventilation and air conditioning system at the Auburn University Beef Teaching Center. Animals were located individually in 10 × 10 foot pens, with nose‐to‐nose contact with their neighbours. Pens were equipped with individual water troughs and feed bunks.

Dietary treatments were based on the transition from a high forage diet to a high concentrate diet (F‐to‐G) and from a high concentrate diet to a high fibre diet (G‐to‐F). Three animals received the F‐to‐G treatment and two animals received the G‐to‐F treatment. Days on treatment for both groups were 64 days in total. The study began with a 14‐day feeding period to allow the steers to adapt to their treatment diets.

The steers assigned to F‐to‐G treatment received from day 1 to 16 a diet consisting of 80% forage and 20% grain (F:G = 80:20); from day 17 to 32, a F:G = 60:40 diet; from day 33 to 48 a F:G = 40:60 diet; and from day 49 to 64 a F:G = 20:80 diet. The steers assigned to the G‐to‐F treatment received from day 1 to 16 a F:G = 40:60 diet; from day 17 to 32 a F:G = 60:40 diet; from day 33 to 48 a F:G = 80:20 diet; and from day 49 to 64 a F:G = 100:0 diet.

Diets were formulated to meet animals’ nutritional needs based on the NRC recommendations for beef cattle (National Academies of Sciences Engineering and Medicine (U.S.). Committee on Nutrient Requirements of Beef Cattle, 2016). The concentrate mix, as‐fed, consisted of 14.18% distillers grains, 49.64% cracked corn, 18.44% dry cottonseed hulls, 10.64% dry soybean hulls, 4.96% molasses, 1.42% A&M 8%‐phosphorous and 0.71% 38%‐limestone. The forage used was Bermuda grass hay. On an as‐fed basis, the Bermuda grass hay had 92.63% DM, 9.83% CP, 32.56% ADF, 61.70% NDF, 4.63% lignin and 55.96% TDN. All cattle had free access to a trace mineral salt block, consisting of 96% NaCl, 2,400 ppm Mn, 2,400 ppm Fe, 260 ppm Cu, 320 ppm Zn, 70 ppm I and, 40 ppm Co (American Stockman^®^ Big 6 Trace Mineral Block).

Rumen epithelium biopsies were performed before changing diets, (i.e. days 17, 33, 49 and 64 days on treatment). In other words, the first biopsy was taken after F‐to‐G steers were receiving a F:G = 80:20 for 16 days and G‐to‐F steers were receiving a 40:60 diet for 16 days. Second biopsy was taken after steers from F‐to‐G treatment were on 60:40 diet for 16 days and when steers from G‐to‐F treatment were on a 60:40 diet for 16 days. Third biopsy was taken after F‐to‐G steers were on a 40:60 diet for 16 days and when G‐to‐F steers were on a 80:20 diet for 16 days. The fourth biopsy was taken when F‐to‐G steers were on a 20:80 diet for 16 days and G‐to‐F steers were on a 100:0 diet for 16 days. The biopsies were performed under field conditions at each animal's individual pen prior to the morning feeding. Instruments for the surgical procedures were sterilized by autoclaving. Surgical scissors and forceps were placed in chlorhexidine diacetate (Nolvasan, Zoetis Animal Health) prior to ruminal biopsy and rinsed with saline solution before harvesting the tissue. Prior to biopsy, the rumen was partially emptied. Rumen contents were kept in a covered bucket and were returned to the rumen immediately after the biopsy. Once partially empty, the ventral sac of the rumen was retracted up to the cannula opening where papillae were excised using surgical scissors. Feed particles were rinsed away with water prior to harvesting epithelium. For each biopsy, approximately 0.5 g of papillae (wet weight) and an additional five individual papillae from each steer were excised. Additionally, samples of rumen fluid were collected in polypropylene tubes and the rumen pH was taken immediately after collection using a calibrated pH meter (VWR SympHony BioP).

### 2.2 Rumen papillae histology

The rumen papillae histological analysis followed a protocol previously described (Ragionieri et al., 2016). Briefly, three papillae samples from each animal at each time point were immersed in 10% neutral buffered formaldehyde for at least 5 days. Then, the samples were dehydrated and processed using a Spin Tissue Processor Microm STP‐120 (Thermo Scientific) according to manufacturer instructions. Following dehydration, the papillae were individually imbedded in paraffin wax. Histological sections (4 μm thick) were then stained with haematoxylin and eosin for morphometric analysis under a light microscope (Nikon Eclipse TS100 microscope, TS 100LED‐F‐MV) equipped with a digital CCD camera (Nikon Fi2 camera and DS‐U3 controller). Digital images were captured, and papillae length (distance between the base and the tip of the papillae) and width (at the middle of the papillae) were measured in random order using Nikon Elements^®^ software.

### 2.3 RNA extraction

Using a bead beater, 50 mg of rumen epithelium from each animal at each collection date was homogenized for 30 s with 1 ml of Qiazol solution (Qiagen miRNeasy Mini Kit; Cat. number: 217004), cooled on ice for one minute and then homogenized again for another 30 s. The samples were then centrifuged for 10 min at 11304 *g* at 4°C to remove insoluble material. The homogenized portion was transferred to a new tube and allowed to sit at room temperature for 5 min. Then, 200 µl of chloroform was added and each sample tube was shaken for 15 s. The samples sat for 3 min at room temperature, followed by centrifugation for 15 min at 11304 *g* at 4°C. The upper phase containing the RNA was transferred to a new tube and 750 µl of 100% ethanol were mixed. The total extracted RNA was cleaned following Qiagen miRNeasy Mini Kit Cat. number 217004 manufacturer's protocol. The concentration of extracted RNA was measured using a NanoDrop One C spectrometer instrument (Thermo Fisher).

### 2.4 Primer design

The cDNA sequence for each gene of interest was retrieved from the University of California‐Santa Cruz's Genome Browser (https://genome.ucsc.edu/) or National Center for Biotechnology Information (NCBI; https://www.ncbi.nlm.nih.gov/). Each gene sequence was imported into Primer Express 3.0.1 software (ABI). The default settings (TaqMan^®^ MGB quantification) were used, except for the minimum amplicon size that was adjusted to 100 base pairs. The Basic Local Alignment Search Tool (BLAST) from the NCBI webpage for *Bos Taurus* database was used with default setting, to test the designed primer sequences and ordered from Integrated DNA Technologies (https://www.idtdna.com). Primer's information can be found in Table S1.

### 2.5 cDNA synthesis

The total RNA was diluted to a concentration of 100 ng/μl. Then, Master Mix 1 (MM1) was prepared by mixing 9 µl of RNase‐free water to 1 µl of Random Primers (Roche Diagnostics). Then, 1 µl of 100 ng total RNA was added. The mixture was incubated at 65°C for 5 min. After this first incubation, the samples were kept on ice for 3 min. For each sample, Master Mix 2 (MM2) was prepared by mixing 1.625 µl RNase‐free water, 4 µl 5× first‐strand buffer, 1 µl Oligo dT18, 2 µl 10 mM dNTP mix (10 mM), 0.25 µl of Revert aid (200 U/µl), and 0.125 µl of RNase inhibitor (20 U/µl). Finally, MM2 was added to MM1 + RNA (final volume 20 µl per gene), and tubes were incubated using the following temporal profile: 25°C for 5 min, 42°C for 60 min and 70°C for 5 min followed by 4°C. Each sample was run in triplicate, and a seven‐point relative standard curve plus the non‐template control were used for quantitative analysis (User Bulletin no. 2; Applied Biosystems).

### 2.6 Preliminary primer testing

In a PCR tube, 8 µl of pooled cDNA, 10 µl of Perfecta SYBR Green, 1 µl of forward primer and 1 µl of reverse primer for each tested gene was added. The samples were placed in an Eppendorf nexus gradient thermocycler for 2 min at 50°C, 10 min at 95°C, 40 cycles of 15 s at 95°C and 1 min at 60°C (denaturation). Five µl of the PCR product was transferred into a new 0.2 ml PCR tube for agarose gel electrophoresis analysis, and 2 µl of loading dye was added. The ladder was prepared by vortexing together 0.6 µl of ladder (25 bp, from Invitrogen) with 2 µl of loading dye. Three grams of agarose was dissolved in 150 ml of 1× TAE buffer. Two µl of SYBR Safe was added, and then, the solution was loaded into the agarose gel apparatus. The ladder was added to one column of each row, and the samples were added to their corresponding wells. The gel ran at 80 mV until the samples had made it ¾ of the way across the gel. The gel was then analysed in a Bio‐Rad Chemi‐Doc apparatus using Image Lab software. PCR products from tested primers with a single, clear band at 100 bp were deemed acceptable.

The manufacturer protocol for the QIAquick^®^ PCR Purification Kit (Qiagen) was used to clean PCR product before sending for sequencing at the University of Illinois Core Sequencing facility. The sequencing results were searched against the *Bos Taurus* database using NCBI Nucleotide BLAST and default settings. Only sequencing results that matched the primer's sequence at a 100% level of similarity were used (Table S3). Genes selected for transcript profiling in the present study were grouped as follows: cellular membrane structure, claudin 1 (*CLDN 1*), claudin 4 (*CLDN4*) and desmoglein 1 (*DSG1*); fatty acid metabolism, acyl‐CoA dehydrogenase short/branched chain (*ACADSB*) and carnitine palmitoyltransferase 1A (*CPT1A*); glycolysis, phosphofructokinase, liver type (*PFKL*); ketogenesis, 3‐hydroxymethyl‐3‐methylglutaryl‐CoA lyase (*HMGCL*), 3‐hydroxy‐3‐methylglutaryl‐CoA synthase 2 (*HMGCS2*), acetyl‐CoA acetyltransferase 1 (*ACAT1*); lactate/pyruvate, lactate dehydrogenase A (*LDHA*); oxidative stress NAD(P)H dehydrogenase quinone (*NQO1*); tissue growth and structure, AKT serine/threonine kinase 3 (*AKT3*), epidermal growth factor receptor (*EGFR*), epiregulin (*EREG*), insulin‐like growth factor‐binding protein 5 (*IGFBP5*) and insulin receptor substrate 1 (*IRS1*); and the urea cycle, solute carrier family 14 member 1 (*SLC14A1*) (Tables S1–S3).

### 2.7 RT‐PCR

Four µl diluted cDNA sample, negative controls and standard curve were pipetted into their respective wells in a MicroAmp™ Optical 96‐well reaction plate in triplicate. Then, 12 µl of SYBR Green Master Mix was placed in each well. The PCR reaction was performed in an ABI Prism 7500 HT SDS instrument with the following conditions: 2 min at 50°C, 10 min at 95°C and 40 cycles of 15 s at 95°C followed by 1 min at 60°C. The RT‐qPCR data were then analysed using the 7500 HT Sequence Detection Systems Software (version 2.2.1, Applied Biosystems). Before statistical analysis, RT‐qPCR data were normalized using the geometric mean of the internal control genes, ubiquitously expressed prefoldin‐like chaperone (*UXT*) and glyceraldehyde 3‐phosphate dehydrogenase (*GAPDH*).

### 2.8 Statistical analysis

Response variables including steer traits (steer body weight, feed intake, papillary length and width and rumen pH) and normalized gene expression levels were described using a linear mixed effects model. Fixed effects included treatment (levels: F‐to‐G or G‐to‐F), day when rumen was biopsied and gene expression levels profiled (days 17, 33, 49 and 64), and interaction. The longitudinal nature of the experimental design and individualized diet changes within treatment was modelled using a repeated structure with an unstructured variance–covariance structure and steer as subject unit.

Of particular interest in the present study was the evaluation of the impact of the particular combination of diet and day during the trial on the steer growth, feed intake, histological and gene expression variables studied. This factor corresponded to the treatment‐by‐day interaction included in the model used to describe the animal performance, histological and gene expression measurements. Also of interest was the assessment of the impact of the overall treatment (F‐to‐G or G‐to‐F) on the previous measurements. The day term allowed us to adjust the interaction estimates for potential trial day‐to‐day differences. These differences encompass differences in particular diets used at each day and differences between sampling days. The composite nature of the effects accounted for by the day term offers less information, and therefore, the results and discussion focus on the interaction and treatment effects.

The model terms were tested including a Kenward–Rogers adjustment of degrees of freedom. The analysis was implemented using the MIXED procedure in SAS (SAS v9.4, SAS Institute). Significant differences were declared at *p* ≤ .05 and tendencies between 0.05 ≤ *p* ≥ .1.

## 3 RESULTS

### 3.1 Animal performance and histological analysis

A tendency for a significant treatment × day on trial interaction for body weight (*p* = .08) and a day effect (*p* < .01) was observed, but there was not a treatment effect (*p* = .19) (Figure S1). In addition, a non‐significant treatment × day interaction for ruminal pH (*p* = .15) was observed, with no significant day effect (*p* = .17) and treatment effect (*p* = .26). Throughout the study, rumen fluid pH remained within 6.8 and 7.3 (Figure S2). Feed intake data had a significant treatment × day interaction (*p* < .01), a treatment effect (*p* = .03) and a day effect (*p* < .01) (Figure S3). Furthermore, there was not a significant treatment × day interaction (*p* = .17 and *p* = .26), treatment effect (*p* = .38 and *p* = .73) or day effect (*p* = .33 and *p* = .15) for rumen papillae length and width respectively (Figure S4).

### 3.2 Gene expression

Table S2 depicts the RT‐qPCR performance of all genes analysed. Relative mRNA abundance, which is the individual proportion of mRNA present in the rumen tissue compared to the abundance of all genes analysed, showed that overall, *LDHA* had the greatest relative mRNA abundance (36%) and *IRS1*, the lowest (0.01%) (Table S2 and Figure S5).

#### 3.2.1 Cell membrane structure

**Figure 1:**
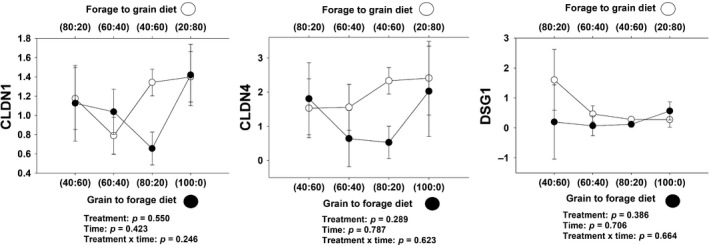
Expression of genes related to cell membrane structure in rumen epithelium papillae from forage to grain treatment and from grain to forage treatment

There was not significant treatment × day interaction for *CLDN1* (*p* = .25) or CLDN4 (*p* = .62) (Table 1 and Figure 1). Furthermore, there was not a significant treatment × day interaction for *DSG1* (*p* = .66). However, relative mRNA abundance of *CLDN1* was greater (2.68%) as compared to *CLDN4* and *DSG1* with 0.87 and 0.09% respectively (Table S2).

**Table 1:** Overall least mean squares values for expression of genes analyzed in rumen epithelium papillae of Jersey steers from forage to grain treatment and from grain to forage treatment

Genes	Treatments		*SEM* *	*p* values	
	F‐to‐G	G‐to‐F		Treatment	Treatment × Day
ACADSB	1.29	0.74	0.10	.0019	.0959
ACAT1	1.07	0.73	0.09	.0171	.9580
AKT3	0.98	1.26	0.16	.2058	.0747
CLDN1	1.18	1.06	0.15	.5503	.2463
CLDN4	1.96	1.25	0.48	.2891	.6226
CPT1A	0.71	0.78	0.11	.6385	.9776
DSG1	0.66	0.24	0.33	.3855	.6643
EGFR	1.18	0.93	0.15	.2843	.1262
EREG	1.07	1.01	0.23	.8479	.7806
HMGCL	1.12	0.92	0.06	.0252	.4144
HMGCS2	1.23	0.72	0.11	.0060	.5563
IGFBP5	1.24	0.93	0.15	.1559	.0777
IRS1	1.15	0.72	0.17	.0837	.4487
LDHA	0.81	0.95	0.04	.0276	.3604
NQO1	1.26	0.56	0.16	.0083	.9285
PFKL	0.97	1.19	0.07	.0420	.1769
SLC14A1	1.03	0.62	0.16	.0969	.9564

* Standard Error of the Mean.

#### 3.2.2 Fatty acid metabolism

**Figure 2:**
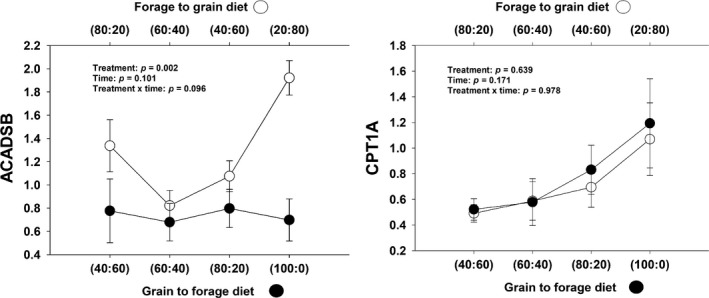
Expression of genes related to fatty acid metabolism in rumen epithelium papillae from forage to grain treatment and from grain to forage treatment

There was a tendency of a significant treatment × day interaction for *ACADSB* (*p* = .09) and a treatment effect (*p* = .002) (Table 1). In F‐to‐G steers, *ACADSB* expression decreased at the beginning of the study followed by a consistent increase in expression with increase of grain (Figure 2). In G‐to‐F steers, there was consistent *ACADSB* expression throughout the study. Additionally, a non‐significant statistical difference for *CPT1A* expression was observed (*p* = .98) (Table 1, Figure 2).

#### 3.2.3 Glycolysis

**Figure 3:**
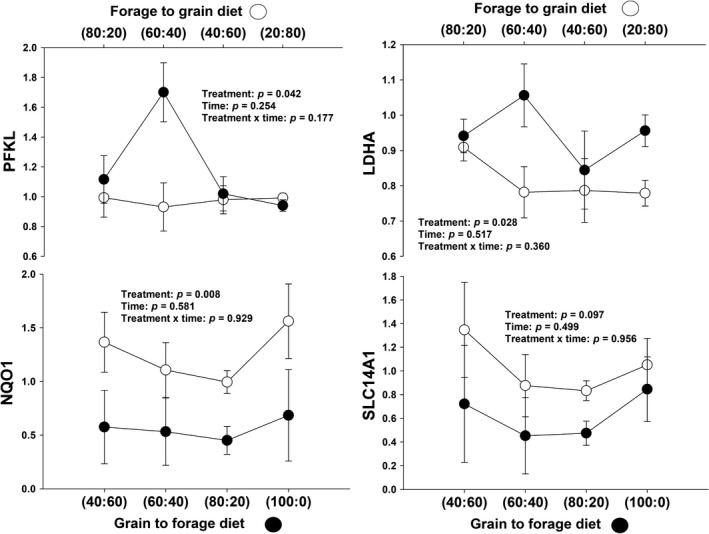
Expression of genes related to glycolysis (PFKL), lactate/pyruvate (LHDA), oxidative stress (NQO1) and urea cycle (SLC14A1) in rumen epithelium papillae from forage to grain treatment and from grain to forage treatment

There was not a significant treatment × day interaction (*p* = .18) but a significant treatment effect for *PFKL* (*p* = .04; Table 1). In G‐to‐F steers, *PFKL* expression increased between the changes from a 40:60 diet to a 60:40 diet. Then, expression decreased when offered the 80:20 diet. However, *PFKL* expression did not present significant changes for F‐to‐G treatment. There was a significant difference in *PFKL* expression between treatments after being fed the 60:40 diet for 16 days (Figure 3).

#### 3.2.4 Pyruvate/lactate metabolism

There was not a significant treatment × day interaction (*p* = .36) but a significant treatment effect (*p* = .03) for *LDHA* (Table 1). In G‐to‐F steers, *LDHA* expression was inconsistent with slight increased and decreased regulation throughout the days evaluated. There was a difference in *LDHA* expression between treatments after both groups being under a 60:40 diet for 16 days and at the end of the study (Figure 3).

#### 3.2.5 Oxidative stress

A non‐significant treatment × day interaction (*p* = .93) was observed for *NQO1* gene expression, but a significant treatment effect (*p* = .01) (Table 1 and Figure 3).

#### 3.2.6 Urea cycle

A non‐significant treatment × day interaction for *SLC14A1* gene expression (*p* = .96) was observed, although there was a tendency for a significant treatment effect (*p* = .09) for *SLC14A1* gene expression (Table 1 and Figure 3).

#### 3.2.7 Ketogenesis

**Figure 4:**
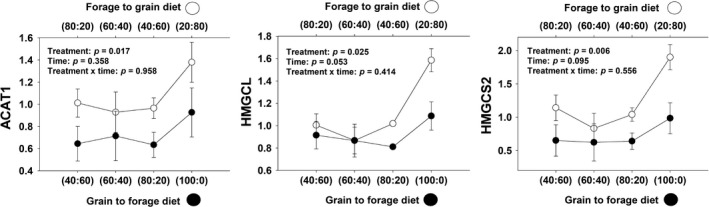
Expression of genes related to ketogenesis in rumen epithelium papillae from forage to grain treatment and from grain to forage treatment

A non‐significant treatment × day interaction (*p* = .96) was observed for *ACAT1* gene expression, but a significant treatment effect (*p* = .02) (Figure 4).

A non‐significant treatment × day interaction (*p* = .41) but a significant treatment effect (*p* = .02) and a day effect (*p* = .05) was observed for *HMGCL* gene expression (Table 1 and Figure 4). Furthermore, a non‐significant treatment × day interaction (*p* = .56) but a tendency for a significant day effect (*p* = .09) and a treatment effect (*p* = .006) was observed for *HMGCS2* gene expression (Table 1 and Figure 4).

#### 3.2.8 Tissue growth/development

**Figure 5:**
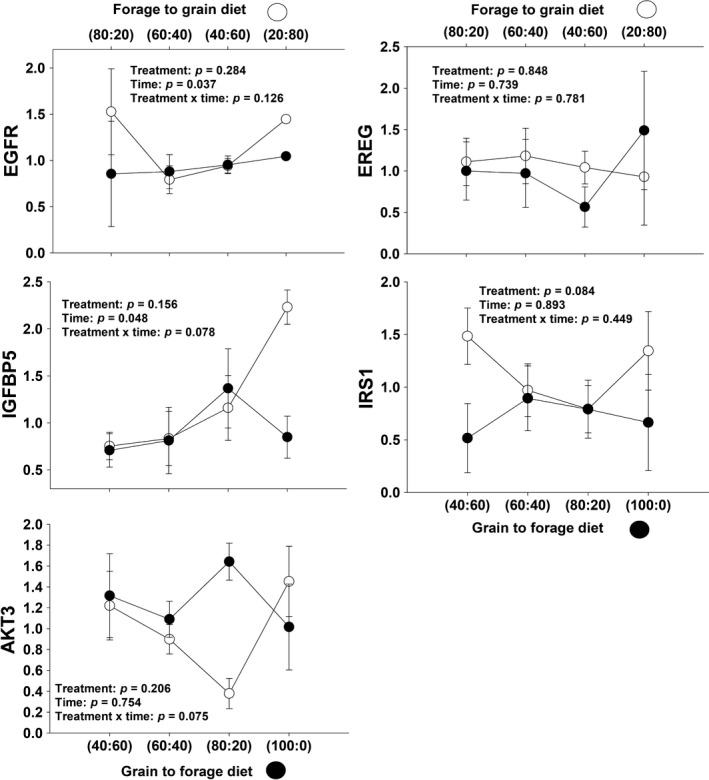
Expression of genes related to tissue growth and development in rumen epithelium papillae from forage to grain treatment and from grain to forage treatment

A tendency for a significant treatment × day interaction (*p* = .07) was detected for *AKT3* gene expression (Table 1). In F‐to‐G treatment, there was a significant decrease of *AKT3* expression between the change from a 60:40 diet to a 40:60 diet and there was a significant increase after receiving the 20:80 diet for 16 days. In G‐to‐F treatment, *AKT3* expression had a significant increase between the 60:40 and the 80:20 diet change and a decrease between 80:20 and 100:0 diets. There was a significant difference in *AKT3* expression between treatments after the time of the third biopsy day (Figure 5).

A non‐significant treatment × day interaction (*p* = .13) but a significant day effect (*p* = .04) was observed for *EGFR* (Table 1), although there was a significant difference in *EGFR* gene expression between treatments at the end of the study (Figure 5). Furthermore, a non‐significant treatment × day interaction was observed for *EREG* gene expression (*p* = .78) with no treatment or day effect (*p* > .05; Figure 5).

A tendency for a significant treatment × day interaction (*p* = .08) and a day effect (*p* = .05) was observed for *IGFBP5* gene expression (Table 1). In F‐to‐G treatment, *IGFBP5* expression generally increased with the addition of grain in the diet, especially during the last 16 days of study when under a 20:80 diet, although for G‐to‐F treatment, *IGFBP5* expression did not have a clear pattern with days on study (Figure 5).

Finally, a non‐significant treatment × day interaction (*p* = .45) but a significant treatment effect (*p* = .08) was observed for *IRS1* gene expression (Figure 5). At the beginning of the study, F‐to‐G treatment had greater *IRS1* expression compared to G‐to‐F treatment (Table 1).

## 4 DISCUSSION

### 4.1 Animal performance

Steers in F‐to‐G treatment exhibited a playing behaviour with the hay at the beginning of the study that often resulted in the hay being out of the reach of the steer, producing a significant amount of feed refusal. This undesired behaviour was solved when steers started to be fed twice daily and feed refusal was measured. This explains the increment in body weight observed between a 14‐day period of adaptation to the treatment diets and the first dietary change at 16 days from the beginning of the study (Figure S1). A total of 9 kg of feed was offered once a day at the beginning of the study. First, steers were receiving the same amount of feed independently of the proportion of grain in the diet. After observing the undesired eating behaviour, animals were fed twice per day decreasing the playing behaviour. Feed intake data were calculated based on the amount of feed offered minus amount of feed refused (Figure S3). Lastly, the rumen pH was within healthy limits throughout all the study in accordance with a similar study, despite a change in body weight and diet composition (Lancaster et al., 2015). However, we were expecting significant alterations in rumen pH due to changes on the diet. Apparently, the progressive change between 16 days, adding or removing 20% of the grain portion of the diet, provided time enough to the rumen microflora to adapt to the new environmental conditions.

### 4.2 Histology

Rumen papillae morphology is key to digestive physiology, since they increment the surface area for absorption of nutrients (Dieho et al., 2016). More efficient cattle have a thicker rumen epithelium compared to their less efficient counterparts (Lam et al., 2017). Furthermore, rumen papillae density is affected by diet (Jing et al., 2018). Introduction of forage will cause papillae to become shorter (Castells, Bach, Aris, & Terre, 2013). Even early on, cattle fed high concentrate diets develop longer papillae which lead to better beef quality (Reddy et al., 2017). However, our study did not show similar results as those mentioned above. Rumen papillae length and width did not present significant changes due to variations in grain proportions in the animal's diet.

### 4.3 Rumen epithelium gene expression

Although the rumen's adaptation to various diets has been extensively studied, research on how rumen epithelium genes are being affected by this change is still in its infancy. Identification of gene responses driving these changes may enhance our precision to formulate diets and better transition cattle from the pasture‐based systems to a feedlot. Following, gene clusters selected for this study will be discussed focusing on their role on the rumen papillae metabolism.

### 4.4 Cell membrane structure

Cell membrane structure and its integrity are fundamental for cell survival. Selected genes associated with membrane structure include claudin‐1 (*CLDN1*), claudin‐4 (*CLDN4*), and desmoglein 1 (*DSG1*). Claudins are associated with endothelial tight junction components (Collins et al., 2006), and they are primarily expressed in the cells of the stratum granulosum (Graham & Simmons, 2005). Loss of surface cells would cause *CLDN1* to be activated (Liu, Xu, Liu, Zhu, & Mao, 2013). *Claudin‐1* activation could also represent an adaptive response to prevent a reduction in permeability during low feed intake (Pederzolli et al., 2018). In lactating Holstein cows, both *CLDN1* and *CLDN4* expression were upregulated in rumen epithelium with the lower pH of induced subacute ruminal acidosis (SARA). However, no mechanism for this upregulation has been found (McCann et al., 2016). Furthermore, *CLDN4* expression was significantly increased in the intestinal mucosa lesion of mice (Tamura et al., 2008). Our results cannot be compared with previous studies because only moderate pH variations under healthy limits were detected (Figure S2). Furthermore, there was a lack of significant differences in gene expression due to treatment and days in trial on these genes (Table 1 and Figure 1).

Desmosomes are structures by which two adjacent cells are attached, formed from protein plaques in the cell membranes linked by filaments (Garrod, 1986). Desmosomes are composed of desmosome–intermediate filament complexes that can be broken into three regions: the extracellular core region, or desmoglea, the outer dense plaque and the inner dense plaque (Delva, Tucker, Kowalczyk, & Andrew, 2009). The outer dense plaque contains the intracellular ends of desmocollin and desmoglein; this last one being the structure, we were interested in analysing. Desmoglein (*DSG1*) is a cell structure involved in cell‐to‐cell adhesion, and it is associated with cellular transport and structure through dynamic changes through ion bindings (Wang et al., 2017). *DSG1* is highly expressed in stratified squamous epithelium, especially the stratum granulosum (Green & Simpson, 2007). Previous studies have shown disruption of tight junctions when cattle were switched to grain‐based diets and developed SARA (Steele, Croom, et al., 2011). It is hypothesized either increased permeability or paracellular transport may be one of the mechanisms by which short‐chain fatty acids (SCFA) are cleared from the rumen in response to the increased organic acid load imposed on the ruminal environment during SARA (Steele, Croom, et al., 2011). Furthermore, *DSG1* was downregulated with high amounts of grain (Steele, Croom, et al., 2011). Further, the expression of *DSG1* was found to be upregulated in the rumen epithelium of cattle recovering from an acidotic bout (McCann et al., 2016). In the rumen epithelium of cattle fed different proportions of grain in the diet, *DSG1* expression was not affected significantly (Figure 1).

### 4.5 Fatty acids

Lipids are hydrolysed into fatty acids in the rumen and later absorbed in the small intestine (Harfoot, 1978). The extent of hydrolysis depends upon rumen pH and may therefore vary between diets (Gerson, John, & King, 1985). Fatty acids are an important source of energy in ruminants. Two examples of the genes linked to fatty acid regulation include Acyl‐CoA dehydrogenase short/branched chain (*ACADSB*) and carnitine palmitoyltransferase 1A (*CPT1A*).

*ACADSB* is associated with short/branched‐chain FA metabolism (Rozen et al., 1994), more specifically the first step of the mitochondrial β‐oxidation reaction (Ensenauer et al., 2005). However, it was differentially expressed in bovine mammary epithelium (Jiang et al., 2018). *ACADSB* was more highly expressed in cows whose milk had a high milk fat content compared to those with lower milk fat (Jiang et al., 2018). In F‐to‐G treatment steers, *ACADSB* gene expression increased sharply at the end of the study; possibly, the delay in increased expression may be related to a threshold of FAs not met until the highest grain diet was provided. Our results for *ACADSB* may indicate that ruminal epithelium is likely more reliant on oxidation of short‐ and branched‐chain FA to derive energy because this gene accounted for almost 9% of total mRNA measured (Table S2).

*CPT1A* catalyses the entry of long‐chain fatty acids (LCFA) into mitochondria and the first step of mitochondrial β‐oxidation of LCFA (Zammit, 1984). *CPT1A* expression in the rumen epithelium of calves was not affected by varying milk replacer treatments; however, expression was downregulated with time (Naeem, Drackley, Stamey, & Loor, 2012). In rat liver, *CPT1A* transcription increase with increasing levels of glucose, but its translation was not affected (Serviddio et al., 2011). However, with a small increase in non‐esterified fatty acid (NEFA) concentrations (i.e. up to 1.2 mmol/L) transcription and translation of *CPT1A* mRNA was increased, although from 1.2 to 4.8 mmol/L both decreased. Furthermore, this higher concentration of NEFA also reduced fatty acid oxidation (Xu et al., 2011). Nonetheless, our study did not present any treatment or days on trial effect on *CPT1A* gene expression.

### 4.6 Glycolysis

Glycolysis is the process in which glucose is broken down for energy. This produces adenosine triphosphate (ATP) and is the primary energy source for animal cells. Phosphofructokinase, liver type (*PFKL*), is one of the genes associated with this process. *PFKL* catalyses the phosphorylation of fructose 6‐phosphate to fructose 1, 6‐bisphosphate. This irreversible reaction serves as the major rate‐limiting step of glycolysis (Graham et al., 2015). At the end of the period when G‐to‐F treatment steers received the 60:40 diet for 16 days, glycolysis appears to be a crucial compensatory mechanism for a period of increased energy demand, which could be filled by ATP‐generating processes like glycolysis (Laarman, 2015). However, we cannot determine the reason for this *PFKL*’s peak at the specified situation. Finally, *PFKL* had higher expression in low residual feed intake (RFI) cattle, indicating greater energy production. This may convert to *PFKL* gene, in a good candidate gene to select animals for feed efficiency (Kong, Liang, Chen, Stothard, & Guan, 2016).

### 4.7 Lactate/pyruvate metabolism

Pyruvate is an important intermediate in key pathways of energy metabolism. Lactate dehydrogenase A (*LDHA*) is a cytoplasmic enzyme involved in the reversible catalysis of anaerobic glycolysis where L‐lactate and NAD are converted to pyruvate and NADH (Valvona, Fillmore, Nunn, & Pilkington, 2016). *LDHA *levels decreased in calves fed an enhanced plane of nutrition, possibly leading to greater utilization of circulating amino acids for gluconeogenesis by ruminal tissue (Naeem et al., 2014). Increased expression of *LDHA* during nutrient restriction suggests an increase in lactate production. Lactate production occurs when oxygen levels are low, and it can be necessary in order to regenerate NAD+, which is consumed in the synthesis of pyruvate from glucose, ensuring that energy production is maintained (O'Shea, Waters, Keogh, Kelly, & Kenny, 2016). In accordance with this, our results showed that G‐to‐F treatment steers had a decrease in *LDHA* expression when the animals were gaining body weight during the first 16 days under study. Furthermore, in another study, ruminal *LDHA* levels decreased in calves fed an enhanced plane of nutrition, possibly leading to a decrease in lactate production (O'Shea et al., 2016).

### 4.8 Oxidative stress

Oxidative stress is caused by the overabundance of reactive oxygen species (ROS) and antioxidants that produce an imbalance that can lead to cellular damage (Martins, Chubatsu, & Meneghini, 1991). This imbalance occurs between the production of free radicals and the ability of the body to counteract or detoxify their harmful effects through neutralization by antioxidants (Celi, 2011). In dairy cattle, cows with higher β‐hydroxy‐butyric acid (BHBA) and NEFA showed higher reactive oxygen metabolites (ROM) and lower levels of antioxidants. Additionally, cows that had higher body condition score (BCS) and greater BCS losses during the peripartum period were more sensitive to oxidative stress (Bernabucci, Ronchi, Lacetera, & Nardone, 2005). NADPH quinone dehydrogenase 1 (*NQO1*) protects cells from damage caused by oxidative stress by acting as a substrate for the two‐electron transferring flavoenzymes (Sarlauskas et al., 2004). In the rumen papillae of Angus × Hereford steers, *NQO1* had increased expression in low RFI individuals (Kern et al., 2017); hence, *NQO1* could be considered as a marker for feed efficiency. Additionally, in cattle fed grain to the point of SARA, *NQO1* expression was downregulated (Abaker et al., 2017). Our results showed that *NQO1* expression increased with the addition of grain in the diet. Furthermore*, NQO1* had significant activation when there was a significant change on the diet (i.e. when 80% of the diet was grain‐base). Nevertheless, our steers maintained a healthy rumen pH throughout the duration of the study.

### 4.9 Urea cycle

Solute carrier family 14 member 1 (*SLC14A1*) mediates the basolateral cell membrane transport of urea across the rumen epithelium (Abdoun, Stumpff, Rabbani, & Martens, 2010; Stewart et al., 2005). In the rumen, urea is turned into ammonia, which is used by rumen microbes as a nitrogen source. As these microbes turn over and are digested in the intestine, this nitrogen returns to the animal as microbial cell protein (Abdoun, Stumpff, & Martens, 2006). Furthermore, *SLC14A1* was upregulated by solid feed intake; this may be due to urea recycling (Berends, Borne, Rojen, Baal, & Gerrits, 2014). *SLC14A1* is expressed and may transport urea into the rumen (Stewart et al., 2005). It has been demonstrated that *SLC14A1* expression is upregulated in the rumen epithelium of sheep fed a high concentrate diet (Lu et al., 2014). In our results, mRNA expression of *SCL14A1* seems to be decreased when grain proportion of the diet was increased in F‐to‐G treatment steers and increased when grain proportion of the diet was reduced in G‐to‐F treatment steers. These contradictory results do not allow drawing any conclusion.

### 4.10 Ketogenesis

Ketosis occurs when too many ketone bodies accumulate in the blood because of not enough glucose available for energy production, resulting in burning stored fat instead. The main cause of ketosis is low dietary energy intake, usually to the extent of the body being in a starvation state. Starvation causes this response because the liver increases output of ketone bodies due to hormonal (i.e. insulin and glucagon) signals. Metabolism of VFAs leads to the formation of ketone bodies via ketogenesis primarily in the liver and rumen (Heitmann, Dawes, & Sensing, 1987). During ketogenesis, VFAs are converted into acetone, acetoacetate, or BHBA. Although ketogenesis is an integral part of ruminant digestion. Too much build up of ketone bodies can cause problems such as ruminal acidosis or ketosis. Ketosis, which occurs in the rumen epithelium, can reduce these cells' ability to absorb nutrients (Leighton, Nicholas, & Pogson, 1983). Some of the genes associated with ketogenesis include acetyl‐CoA acetyltransferase 1 (*ACAT1*), 3‐hydroxymethyl‐3‐methylglutaryl‐CoA lyase (*HMGCL*) and 3‐hydroxy‐3‐methylglutaryl‐CoA synthase 2 (*HMGCS2*).

*ACAT1* encodes a mitochondrial localized enzyme that catalyses the first step in ketogenesis that consist in the reversible formation of acetoacetyl‐CoA from two molecules of acetyl‐CoA (Chang, Huh, Cadigan, & Chang, 1993). Bulls fed a high concentrate diet increased absorption of acetic acid due to greater *ACAT1* activity in rumen epithelium (Harmon et al., 1991). In Angus × Hereford steers, *ACAT1* trended towards increased expression with increased RFI (Kern et al., 2017). In our study, signs of ketogenesis were noticed by a significant increment in *ACAT1* expression at the end of the study in both treatment groups. Previously, it has been shown that bulls fed a high concentrate diet increased absorption of acetic acid due to greater *ACAT1* activity in rumen epithelium (Harmon et al., 1991); our results for F‐to‐G treatment concur with this statement. It has been noted that *ACAT1* expression decreases late in fattening (Lee et al., 2007), which is contradictory to what we observed with an increase in *ACAT1* expression the last 16 days of the study on animals exposed to a high‐grain diet (F‐to‐G treatment).

During periods of positive energy balance, BHBA is produced primarily via ketogenesis from butyrate within the rumen epithelial cells. Mainly, *HMGCL* contributes to ketogenesis by converting HMG‐CoA to acetoacetate (Lane, Baldwin, & Jesse, 2002). However, *HMGCL* was not associated with butyrate production (Laarman, Sugino, & Oba, 2012). In contrast, *HMGCL* and *HMGCS2* have been associated with changes in VFA levels, particularly butyrate (Wang et al., 2016). *HMGCS2* plays a central role in coordinating ruminal ketogenic flux (Naeem et al., 2012) because it encodes the rate‐limiting enzyme in the synthesis of ketone bodies (Ma et al., 2017).

In the rumen papillae of Angus‐Hereford crossbred heifers, *HMGCL* expression increases with larger amounts of grain in animals’ whose pH increased with the introduction of more grain on the diet (Zhao, Chen, Penner, Oba, & Guan, 2017). In primiparous dairy cows fed diets with different neutral detergent fibre (NDF) to starch ratios, the expression of *HMGCS2* was downregulated with increasing NDF to starch ratio (Ma et al., 2017). In the rumen of sheep, both *HMGCL* and *HMGCS2* were upregulated in lambs fed a starter diet in comparison to those that nursed (Wang et al., 2016). At the end of our study, like Wang et al., 2016, F‐to‐G treatment steers had increased expression of both *HMGCL* and *HMGCS2* as the proportion of grain fed increased, denoting a greater degree of ketogenesis.

### 4.11 Tissue growth/structure

The intake of protein‐ and energy‐rich diets promotes the growth of ruminal tissues by promoting epithelial cell proliferation (Shen et al., 2004). AKT serine/threonine kinase 3 (*AKT3*) regulate cell signalling in response to insulin and growth factors. Calves with enhanced early plane of nutrition initially express lower amounts of *AKT3* before it increased at the end of the mild‐fed period (Naeem et al., 2014). This expression pattern (i.e. an initial decrease followed by an increase) is consistent with our results for F‐to‐G treatment steers.

Epidermal growth factor receptor (*EGFR*) is a glycoprotein that binds to epidermal growth factor leading to cell proliferation (Yarden & Schlessinger, 1987). EGFR is associated with tissue growth and structure. *EGFR* is a focal regulatory gene in the adaptive response of rumen papillae in dairy cattle during early lactation (Steele, Dionissopoulos, AlZahal, Doelman, & McBride, 2012). However, in Holstein calves fed either a calf starter diet with or without hay, rumen epithelium expression of *EGFR* was not significantly different (Kim et al., 2016). The significant increase in expression of *EGFR* in F‐to‐G treatment steers may be attributable to the rumen remodelling with the changes in diet.

Epiregulin (*EREG*) acts as a ligand for both epidermal growth factor receptor (*EGFR*) and the erb‐b2 receptor tyrosine kinase 4 (*ERBB4*). It is believed to be involved in a variety of processes including inflammation, wound healing and cell proliferation (Toyoda, Komurasaki, Uchida, & Morimoto, 1997). In rumen papillae, both *EREG* and *EGFR* expression increased as Holstein cows progressed from day 10 to day 28 in milk, indicating ruminal papillae proliferation (Minuti et al., 2015). In our study, *EREG* expression did not present a clear pattern of response to the change in diets.

Genes that belong to the IGF family play a role in the development of rumen epithelial cells (Shen et al., 2004). An increase in the concentration of butyrate or high level of digestible carbohydrates in the diet increased *IGFBP*’s activation and led to greater proliferation and differentiation of rumen epithelial cells (Ma et al., 2017; Zhang et al., 2017). Expression of insulin‐like growth factor‐binding protein 5 (*IGFBP5*) mRNA was upregulated in the rumen papillae of sheep supplemented with a higher plane of nutrition and in cattle transitioning to a high‐grain diet (Jing et al., 2018; Steele, Croom, et al., 2011; Steele et al., 2012; Xu, Wang, Liu, Zhu, & Mao, 2018). Our results show an increase in *IGFBP5* expression as grain proportion increases in the diet (F‐to‐G treatment) and a not clear pattern when diet decrease in grain proportion (G‐to‐F treatment). *IGFBP5* results suggest that the rumen epithelium cells might present higher degree of proliferation with increments in grain content in the diet.

The IRS proteins are a family of cytoplasmic adaptor proteins that transmit signals from the insulin and IGF‐1 receptors to elicit a cellular response (Shaw, 2011). *IRS‐1* and *IRS‐2* are ubiquitously expressed and are the primary mediators of insulin‐dependent mitogenesis and regulation of glucose metabolism in most cell types (White, 2002). Insulin receptor substrate 1 (*IRS1*) plays a role in signalling from insulin and insulin‐like growth factor‐binding protein 1 (*IGFBP1*) receptors to various intracellular pathways. *IRS1* phosphorylation begins a cascade of events that leads to glucose uptake. This signalling and subsequent uptake of glucose lead to tissue proliferation (Higashi, Sukhanov, Anwar, Shai, & Delafontaine, 2012). In the rumen epithelium of multiparous Holstein cows, *IRS1* increases with a transition to a higher energy and lower straw diet. The authors surmise that this effect may increase insulin sensitivity (Minuti et al., 2015). The reaction of *IRS1* expression to the 20:80 diet on steers from G‐to‐F treatment coincided with previous results but the lack of *IRS1* expression in steers from F‐to‐G treatment cannot be explained.

## 5 CONCLUSION

Rumen epithelium gene expression results indicated that genes related to tissue growth and structure (*AKT3* and *IGFBP5*) and fatty acid metabolism (*ACADSB*) were the most affected by transitioning diets in order to adapt to the new ruminal environment created by the variation in the proportion of grain in the diet. However, no changes in rumen pH and rumen papillae length or width were observed. In conclusion, studies looking further into these genes as well as others deserve more attention.

## CONFLICT OF INTEREST

Author(s) disclose no potential conflicts of interest.

## AUTHORS' CONTRIBUTIONS

S. J. M. involved in conceptualization, formal analysis, funding acquisition, project administration, writing—review & editing. T. P. N. involved in investigation, methodology, writing—original draft, writing—review & editing, J. D. S. involved in histology analysis, writing, review & editing. R. M. S. Jersey steer health care, rumen cannulation surgeries and rumen epithelium biopsies. G. F. A. involved in laboratory work (RT‐qPCR). S. L. R. and B. R. S. involved in statistical analysis. All authors read and approved of the manuscript.
