# Supplementary material for: Jersey steer ruminal papillae histology and nutrigenomics with diet changes
Source: J Anim Physiol Anim Nutr (Berl). 2019 Sep 4;103(6):1694–707. doi: 10.1111/jpn.13189 (PMC6899929; doi:10.1111/jpn.13189)
Supplement: Supplementary file 1 [file JPN-103-1694-s001.docx]

**Supplemental Table 1.** GenBank accession number, gene ID, sequence, starting point and amplicon size (bp) of primers for *Bos taurus* used to analyze gene expression in rumen epithelium by RT-qPCR.

| Accession # | Gene | Forward Primer | F-start | Reverse Primer | R-Start | BP |
| --- | --- | --- | --- | --- | --- | --- |
| NM_001017933.1 | ACADSB | GCGCTGCTACGAAGAAACTTC | 193 | TTGTCGCTTTGAGCTGAGCTT | 292 | 100 |
| NM_001046075.1 | ACAT1 | GCGGAGCGAAGTTATGTATCAAA | 93 | GAGAGGAAAGACTGCCTAGAAAGGA | 192 | 100 |
| XM_019976103.1 | AKT3 | GGGAGAGCTGTTTTTCCATTTG | 731 | ATGTAGATAGTCCAAGGCAGAGACAA | 830 | 100 |
| AB178476.1 | CLDN1 | CCGTTGGCATGAAGTGTATGA | 517 | GCCAGACCTGAAATAAGAAAGATCA | 616 | 100 |
| NM_001014391.2 | CLDN4 | CCCAGCCAGCAACTACGTCTA | 696 | [TCACAGATTGCAGTGAGCTCAGT](https://www.ncbi.nlm.nih.gov/nucleotide/NM_001014391.2?report=genbank&log$=nucltop&blast_rank=8&RID=DYGGN0UB014) | 797 | 102 |
| NM_001304989.1 | CPT1A | AAATCCTGGTGGGCTACCAAT | 809 | TCGCGAAGTAGTTGCTGTTCA | 908 | 100 |
| [NM_174045.1](https://www.ncbi.nlm.nih.gov/nucleotide/27881409?report=genbank&log$=nucltop&blast_rank=17&RID=TNTAEVSP01R) | DSG1 | AAGGTTGTTAAGCCCCTAGATTTTG | 1094 | ATTGCAGTTGCTGTGAGTTTATATTGA | 1215 | 122 |
| XM_592211.8 | EGFR | TCAGACCATTGCCTTTAATTGAGA | 4609 | TGACCTCCAAAGACTTGATATACTTCCT | 4709 | 101 |
| XM_019962926.1 | EREG | CAAAGTCAAAGCAAGGAGAGAGATG | 2586 | GACACCTCAGGGCAGTTGAATT | 2685 | 100 |
| [NM_001034034.2](https://www.ncbi.nlm.nih.gov/nucleotide/NM_001034034.2?report=genbank&log$=nucltop&blast_rank=26&RID=ADT9YDBU015) | GAPDH | CACTCACTCTTCTACCTTCGATGCT | 932 | CCCTGTTGCTGTAGCCAAATTC | 1035 | 104 |
| NM_001075132.1 | HMGCL | GTACGTCTCCTGTGTGCTTGGA | 519 | CTCGTAGCAGCCCATGGAGTA | 618 | 100 |
| NM_001045883.1 | HMGCS2 | TCTGGTTTGGCAGCAAGTTTCT | 1290 | TTCTGCAGGTCTGATGTACTGGAT | 1390 | 101 |
| XM_010802317.1 | IGFBP5 | ATACTAGATGGTAGGTGTTCCTAAAGCA | 1776 | ACTATCAGAGTGTGGATTGATTTCTCA | 1880 | 105 |
| XM_003581871.1 | IRS1 | TGTTGACTGAACTGCACGTTCT | 4268 | CATGTGGCCAGCTAAGTCCTT | 4379 | 112 |
| [BC146210.1](https://www.ncbi.nlm.nih.gov/nucleotide/148878491?report=genbank&log$=nucltop&blast_rank=31&RID=TSGERYG2016) | LDHA | TGTTGTTTCCAATCCAGTCGATAT | 560 | GGAAGCGAGCTGAATCCAGAT | 669 | 110 |
| NM_001034535.1 | NQO1 | CAATCCCGTCATCTCCAGAAA | 242 | TCAGACGGCCTTCTTTATAAGCTAA | 345 | 104 |
| NM_001080244.2 | PFKL | CGAGAAGATGAAGACAGAGATCCA | 1860 | GGAGGAGTAGAGGTTGTACAGGAACT | 1959 | 100 |
| NM_001008666.1 | SLC14A1 | TATGTCCATGACGTGTCCAGTCT | 515 | GTACATTGACAACGCCATATTGAAG | 620 | 106 |
| [NM_001037471.2](https://www.ncbi.nlm.nih.gov/nucleotide/402692131?report=genbank&log$=nucltop&blast_rank=75&RID=TVA9N51V016) | UXT | CTGGCAGAAGCTCTCAAGTTCA | 337 | GGATATGGGCCTTGATATTCATG | 436 | 100 |
|  |  |  |  |  |  |  |
|  |  |  |  |  |  |  |

**Supplemental Table 2.** RT-qPCR performance table among the 17 genes measured in rumen papillae on Jersey steers.

| **Gene** | **Median Ct^1^** | **Median ∆Ct^2^** | **Slope^3^** | **(R^2^)^4^** |  | **Efficiency (%)**^5^ | **relative mRNA abundance^6^** | **1/E∆Ct^7^** | **%** |
| --- | --- | --- | --- | --- | --- | --- | --- | --- | --- |
| **ACADSB** | 19.500 | -0.468 | -2.529 | 0.998 |  | 148.6 | 1.532 | 0.087 | 8.71 |
| **ACAT1** | 21.103 | 1.176 | -2.704 | 0.999 |  | 134.3 | 0.367 | 0.021 | 2.09 |
| **AKT3** | 25.711 | 5.675 | -2.389 | 0.996 |  | 162.2 | 0.004 | 0.000 | 0.02 |
| **CLDN1** | 20.856 | 0.836 | -2.554 | 0.996 |  | 146.3 | 0.471 | 0.027 | 2.68 |
| **CLDN4** | 22.085 | 2.080 | -2.547 | 0.999 |  | 147.0 | 0.153 | 0.009 | 0.87 |
| **CPT1A** | 22.765 | 2.575 | -2.521 | 0.991 |  | 149.3 | 0.095 | 0.005 | 0.54 |
| **DSG1** | 23.883 | 3.931 | -2.174 | 0.996 |  | 188.4 | 0.016 | 0.001 | 0.09 |
| **EGFR** | 21.380 | 1.327 | -2.567 | 0.998 |  | 145.2 | 0.304 | 0.017 | 1.73 |
| **EREG** | 25.651 | 5.634 | -2.549 | 0.993 |  | 146.8 | 0.006 | 0.000 | 0.04 |
| **HMGCL** | 19.442 | -0.525 | -2.540 | 0.998 |  | 147.6 | 1.609 | 0.092 | 9.15 |
| **HMGCS2** | 18.293 | -1.701 | -2.717 | 0.999 |  | 133.4 | 4.228 | 0.240 | 24.04 |
| **IGFBP5** | 19.863 | -0.086 | -2.583 | 0.998 |  | 143.9 | 1.079 | 0.061 | 6.14 |
| **IRS1** | 26.445 | 6.464 | -2.420 | 0.999 |  | 159.0 | 0.002 | 0.000 | 0.01 |
| **LDHA** | 17.818 | -2.185 | -2.727 | 0.998 |  | 132.7 | 6.330 | 0.360 | 36.00 |
| **NQO1** | 24.605 | 4.637 | -2.598 | 0.999 |  | 142.6 | 0.016 | 0.001 | 0.09 |
| **PFKL** | 22.393 | 2.423 | -2.570 | 0.998 |  | 145.0 | 0.114 | 0.006 | 0.649 |
| **SLC14A1** | 19.832 | -0.258 | -2.594 | 0.999 |  | 142.9 | 1.257 | 0.071 | 7.14 |
| **Total** |  |  |  |  |  |  |  | 1.000 | 100 |

^1^ The median is calculated considering all time points and all steers, ^2^ The median of ∆Ct is calculated as [Ct gene – geometrical mean of Ct internal controls] for each time point and each steer, ^3^ Slope of the standard curve, ^4^ R^2^ stands for the coefficient of determination of the standard curve, ^5^ [10^(-1/slope)]-1 x 100, ^6^ relative mRNA abundance = 1/ Efficiency Median ∆Ct, ^7^1/E∆Ct = relative mRNA abundance/∑relative mRNA abundance.

**Supplemental Table 3.** Sequencing results of PCR products from primers of genes designed for this experiment. Best hits using BLASTN (http://www.ncbi.nlm.nih.gov) are shown.

| Gene | SEQUENCE |
| --- | --- |
| ACADSB | GGCCTGTCTTCCGTGAACTCCTTCCTCATGCCCTTAATTTCTTTCTCAGGTCAGGAAGGCCTTCAGGCTTCAAAGGCGGACAAAAAAT |
| ACAT1 | ACGTAGTAGGTTCATAGTAGGTCTATAAGAACACCCCATGTGATCCTTTCTAGGCAGTCTTTCCTCTCAA |
| AKT3 | GCACGGTTCCTCGGAGGCCGCCACAGGTTTTCTATGGTGCAGAAATTTGTCCTCCTGCCCTTTGGACTATCCTACATACGCNCNTTAAG |
| CLDN1 | CGCATGTGCGCGAGAGCACAGAAGAAAGTCCGGAAGTGGCCGTGCTCTTTGGGGGCCGGTAATCTTTCTTATTTCAGGGTCCTGGCAGACC |
| CLDN4 | AGCAGGTACCTTTTAATTTTTTTCTTTCGTCTTTGTTTCTCCCGTGGACTGGAGCTCACTGCAATCTGTGAAA |
| CPT1A | CGTGGGGGGAGGAACATCTCCTCGAGGGAGAGGGCCGCAGTATGGTTGAACAGCAACTACTTCGCGAACCTTTTAGAGGCGTAT |
| DSG1 | GAATATATCGCACTTGTCTTGGTGTTAGAAATAAAGGCTGGAATTTCATCAAATCAATTATGGTCTCAATATAAACTCACAGGCAACCTGCAATAAA |
| EGFR | GGTACAGCCAAAAGGAACAATCCCCCGAATTTGAGTCCTTAAGGAAGTATATCCAAGGTCCTTTGGAGGTCCAA |
| EREG | GGGCATGTGGGGCAGATTTTTTATTTTTCTGAGTCCGTGAGAGAGGATTAGTTAAATTTCAACCTGCCCCTGAGGTGTCAA |
| GAPDH | GGGCGTCAGTCCCTACGACACCTTGTTCAGCTCATTTCCTGGTACGACAATGGAATTTGGCCTACAGGCAACAGGGTA |
| HMGCL | CGCTTAGGGAAACTTCCCGGGCTAAGTCGCTGAGGTCACCAAGAAGCTGGTACTCCCATGGGCTGCTACGAGGAA |
| HMGCS2 | GTCGCTGTGCAGGTCAGCTCTCGGCGTCCCGCTGGAGAGCTGGTATCGCAGTACATCGAGACCGTGCAGAAACTTGCTGCCAAACCAGAA |
| IGFBP5 | GGGGTGTTAATGGAGGTGGGGTGCATATTTTCACTTCCCCATTGCAGGCCCTCTGAGAAATCAATCCACACCTTCTGAATAGGTAATA |
| LDHA | TGCGACTAGTGGCTGTGAGGATAAGTGGGCTTTCCCAAAAACCGGTGGTTAATTGGGAAGGTGGGTTGGCAATCTGGGAATTCAGCTCGCTTTCCAA |
| NQO1 | GCATGATCGTAGGACCCGGGACTTTCAGTATCCTGGCCGAGACTGGTTTTAGCTTATAAAGAAGGCCGTCTCGAAA |
| PFKL | GGCGGCGTGGATCGGGAGTAGAGGTGCCACGGAGCACTACACCACAGAGGTTTCCTGGTACAACCTCTACTCCTCCAGATTTTTTTGGGGGGG |
| SLC14A1 | CGCACCTTACTCGGTGTTGAGCAAATGGGGACCCTGCCCTGTCTTTCACTCCTGCCCCTTTCAATATGGCGGTTGTCAATGGTACAAAAA |
| UXT | GTCTAAACGTCCCCTCCGGGGGCTCAGCGACAACCTTCACCAAGGGACTTCCATTGAATATTCAAGGGCCCATTATTCCAA |
|  |  |
|  |  |

**Supplemental Figure 1.** Jersey steers body weights from forage to grain treatment (n = 3) and steers from grain to forage treatment (n = 2) through the study.


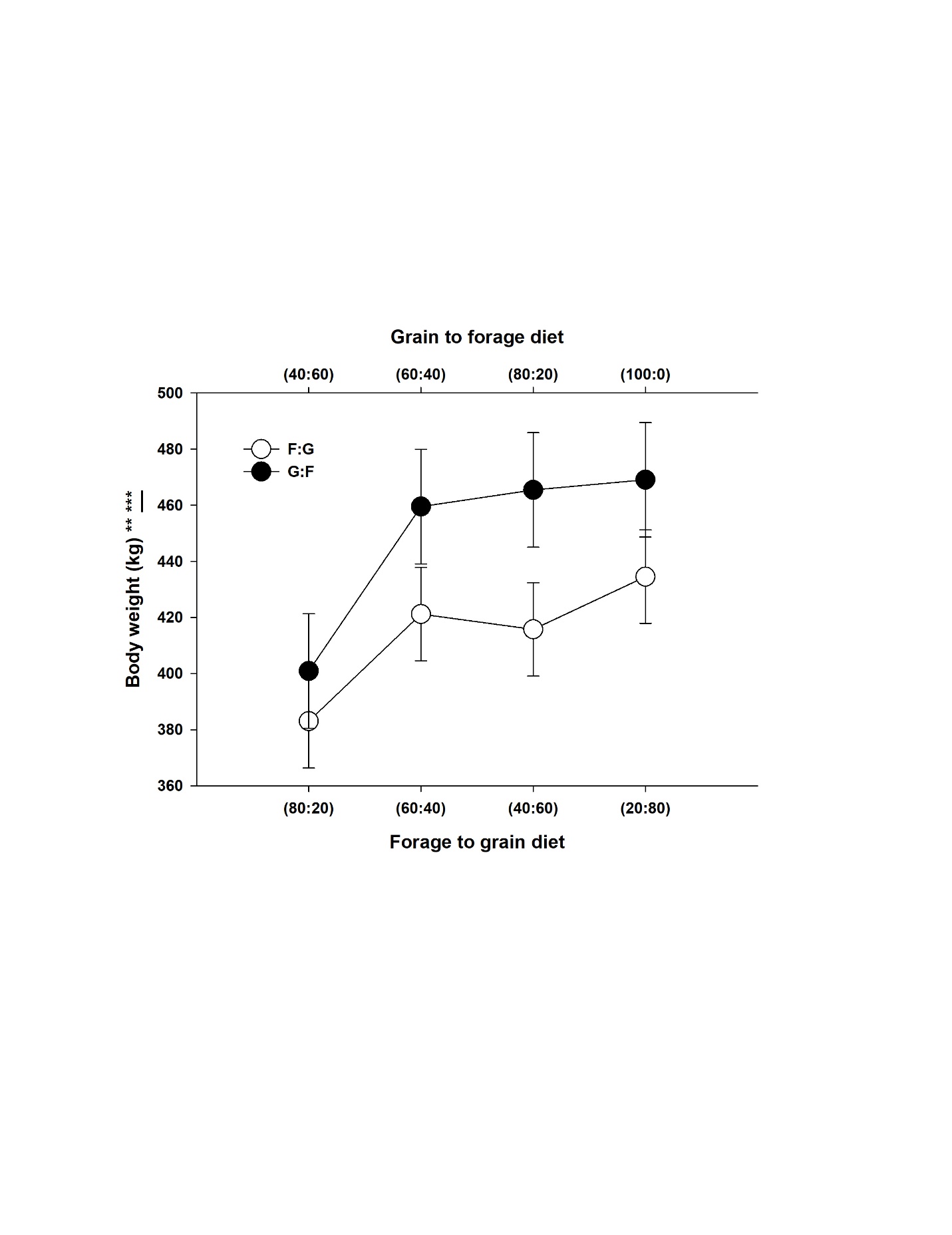


Statistical significant differences where declared at *P* < 0.05 and tendencies at *P* > 0.06 and < 0.1. Treatment × day interaction (***), day effect (**) and treatment effect (*). Symbols (#) on lines denote significant differences (*P* < 0.05) between two time points for the same treatment. Tendencies are denoted if symbols (*, ** or ***) are underlined. Note: First body weight measurement correspond to the beginning of a 21-days adaptation period to treatment diets.

**Supplemental Figure 2.** Jersey steers rumen liquid pH from forage to grain treatment (n = 3) and steers from grain to forage treatment (n = 2) through the study


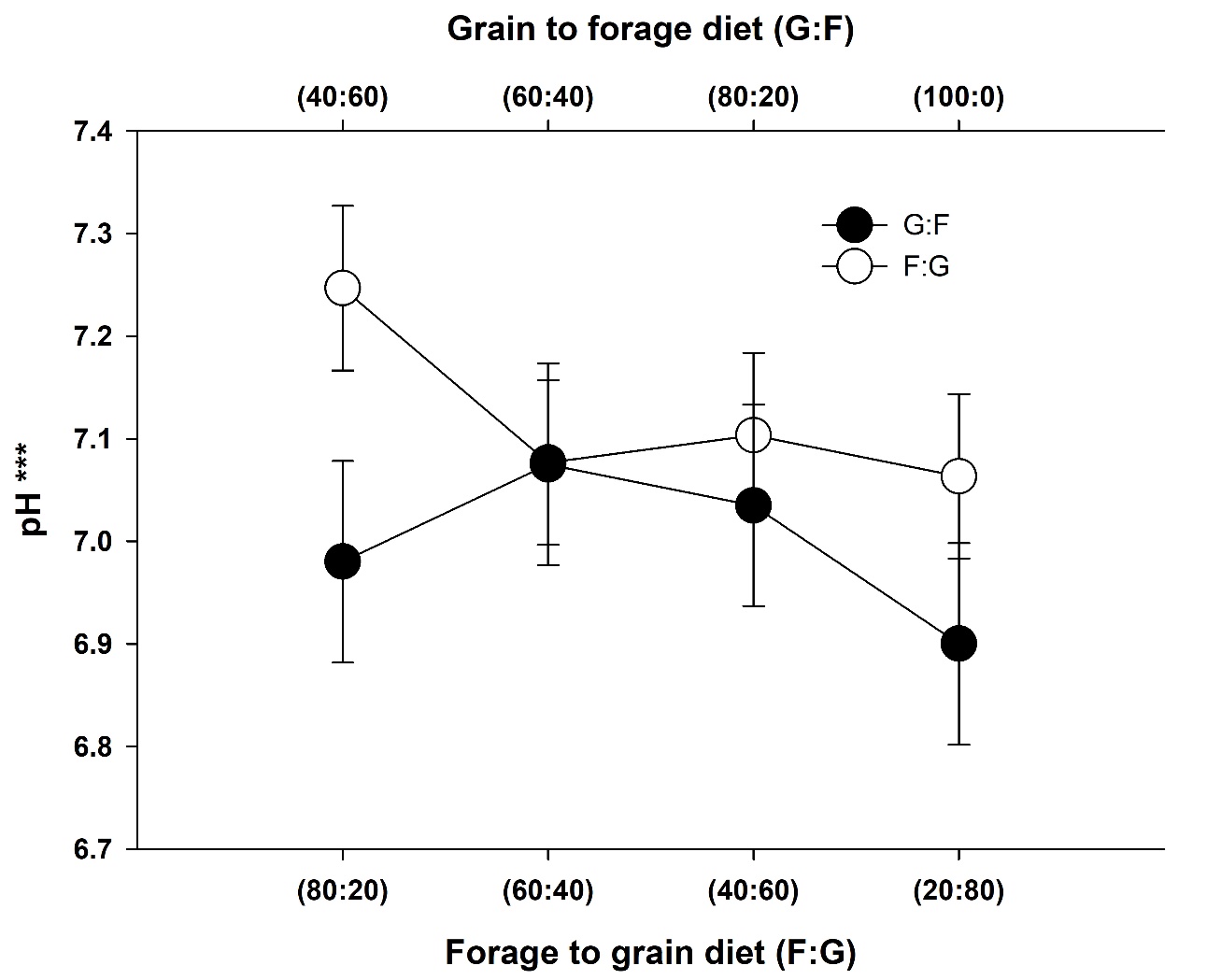


Statistical significant differences where declared at *P* < 0.05 and tendencies at *P* > 0.06 and < 0.1. Treatment × day interaction (***), day effect (**) and treatment effect (*).

**Supplemental Figure 3.** Feed intake throughout the study (64 days) on Jersey steers from forage to grain treatment (F:G) and from grain to forage treatment (G:F).


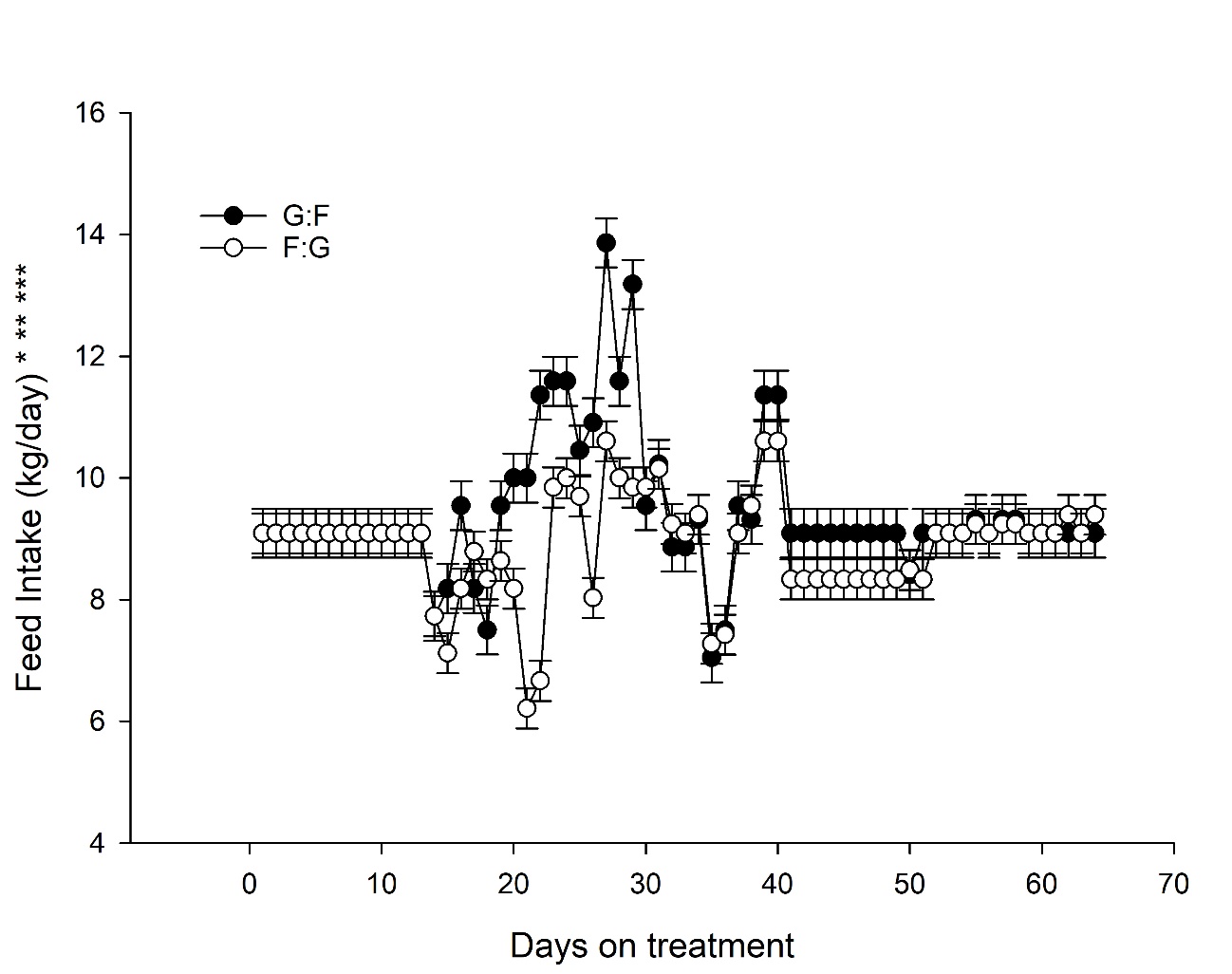


Statistical significant differences where declared at *P* < 0.05 and tendencies at *P* > 0.06 and < 0.1. Treatment × day interaction (***), day effect (**) and treatment effect (*).





**Supplemental Figure 4.** Rumen papillae length and width in Jersey steers from forage to grain treatment and steers from grain to forage treatment

Statistical significant differences where declared at *P* < 0.05 and tendencies at *P* > 0.06 and < 0.1. Treatment × day interaction (***), day effect (**) and treatment effect (*).

**Supplemental Figure 5.** Relative mRNA abundance of genes analyzed in Jersey steers rumen epithelium papillae.

**

**
